# Hunting and Stray Dogs as Reservoirs of *Echinococcus* Species

**DOI:** 10.3390/ani16091421

**Published:** 2026-05-06

**Authors:** Nikola Betić, Milica Kuručki, Katarina Breka, Aleksandra Penezić, Neda Bogdanović, Ilija Pantelić, Ana Vuletić, Tijana Kukurić, Jelena Karanović, Jovana Raičević, Aleksandar Bajčić, Ivana Klun, Aleksandra Uzelac

**Affiliations:** 1 Institute of Meat Hygiene and Technology, 11000 Belgrade, Serbia; nikola.betic@inmes.rs (N.B.); aleksandar.bajcic@inmes.rs (A.B.); 2 Faculty of Biology, University of Belgrade, 11000 Belgrade, Serbia; milicakurucki@yahoo.com (M.K.); katarina.breka@bio.bg.ac.rs (K.B.); aleksandra.penezic@bio.bg.ac.rs (A.P.); neda.bogdanovic@bio.bg.ac.rs (N.B.); ilija.pantelic@bio.bg.ac.rs (I.P.); e3001_2024@stud.bio.bg.ac.rs (A.V.); jovana.raicevic@bio.bg.ac.rs (J.R.); 3 Department of Veterinary Medicine, Faculty of Agriculture, University of Novi Sad, 21000 Novi Sad, Serbia; tijana.kukuric@polj.uns.ac.rs; 4 Institute of Molecular Genetics and Genetic Engineering, University of Belgrade, 11042 Belgrade, Serbia; jelena.karanovic@imgge.bg.ac.rs; 5 Institute for Medical Research, University of Belgrade, 11129 Belgrade, Serbia; iklun@imi.bg.ac.rs

**Keywords:** Cestodae, cyclophyllidea, *Echinococcus multilocularis*, *Echinococcus granulosus sensu lato* complex, tapeworm

## Abstract

In Serbia, human echinococcosis cases are predominantly diagnosed as the cystic form, caused by *Echinococcus granulosus sensu lato* (*s. l.*) complex tapeworms. Alveolar echinococcosis, a more severe disease caused by *Echinococcus multilocularis*, emerged recently. Domestic dogs have been implicated in the transmission of *E. granulosus sensu stricto* G1/3 to humans. In an ongoing screening of definitive hosts for the presence of *Echinococcus* spp. to discover reservoirs of *E. multilocularis*, fecal samples of hunting (n = 29) and stray dogs (n = 66) were examined. Taeniid eggs were collected using flotation and mesh filtration. The presence of Cyclophyllidea, *E. granulosus s. l.* and *E. multilocularis* DNA was detected by specific PCR assays. In total, 60% of the fecal samples contained cyclophyllidean DNA, while *Echinococcus* spp. DNA was detected in 7.4% of the samples. *E. canadensis* G6–8,10 DNA was present in 4.2% and *E. multilocularis* in 3.2% of the samples. *E. multilocularis* DNA was detected only in stray dog feces, suggesting that strays are a reservoir and possibly an important link in the transmission chain. To the best of our knowledge, this study provides the first molecular identification of *Echinococcus* spp. in dogs in Serbia.

## Simple Summary

Tapeworms cause a number of zoonotic foodborne diseases. One such disease, which is of high importance to public health globally, due to difficult and costly treatment and possible fatal outcomes, is echinococcosis. Tapeworm species that cause echinococcosis in Europe include the *Echinococcus granulosus sensu lato* complex and *Echinococcus multilocularis*. The tapeworm’s life cycle consists of definitive and intermediate hosts, usually predator(s) and prey, which are infected after consumption of larvae or eggs, respectively. Domestic dogs are definitive hosts which can be important in the transmission of *Echinococcus* spp. eggs to humans, due to frequent and/or close contact and cohabitation. Dogs whose diet includes raw livestock and/or wildlife meat are most exposed to *Echinococcus* spp. infection. This study analyzed fecal samples of hunting and stray dogs from Serbia and identified DNA of *E. canadensis* G6–8,10 and *E. multilocularis*, which causes the severe alveolar form of echinococcosis. The results are of public health concern and indicate that systematic surveillance of definitive hosts and education to raise awareness of the disease and promote regular deworming should be implemented. Innovative deworming campaigns to specifically target free-roaming stray dogs, which are reservoirs of *E. multilocularis*, may need to be developed in the future.

## 1. Introduction

The class Cestoda consists of different species of tapeworms, some of which are parasites of the gastrointestinal (GI) tract of dogs. Several tapeworm species found in dogs are zoonotic, but comparatively few, such as *Taenia multiceps*, *T. crassiceps* and *T. serialis*, *Echinococcus granulosus sensu lato* (*s. l.*) complex, and *E. multilocularis*, cause infections ensuing from accidental ingestion of parasite eggs found in feces, or from the consumption of infected dog tissue (as with *Spirometra* spp.), and which can result in severe symptoms and/or disease in humans. Tapeworm infections in dogs result from the ingestion of infected primary or secondary intermediate hosts, which include common ectoparasites, such as fleas (for *Dipylidium caninum*), and the tissue of infected amphibians, reptiles, and crustaceans (for *Spirometra* spp.) or mammals and birds (for Taeniidae, including *Echinococcus* spp.; *Mesocestoides* spp.) [1]. Risk factors for infection identified by the European Scientific Counsel Companion Animal Parasites (ESCCAP) and various scientific studies include lifestyle (e.g., pet, hunting, shelter, stray), time spent outdoors, and diet. Additional important factors are hunting behavior and contact with other animals (including wildlife) and especially the status, practice, and frequency of ecto- and endoparasite treatment [2,3,4,5,6].

The most clinically significant species of tapeworms in Europe which may be transmitted to humans by dogs are the *Echinococcus granulosus s. l.* complex and *Echinococcus multilocularis*. The resulting disease, echinococcosis, manifested as either cystic (CE) or alveolar (AE), is hard to diagnose, difficult to cure, and may be fatal (primarily AE) [7]. *Echinococcus* spp. tapeworms have a complex two-host life cycle, with wild and domestic ungulates and small mammals acting as intermediate hosts and Canidae and Felidae as definitive hosts [8]. The maintenance of *Echinococcus* spp. tapeworms in the environment depends on cycling between predator(s) and prey, while aberrant or dead-end hosts, like humans, are not directly involved in transmission [2,8]. Disease develops primarily in intermediate/aberrant hosts, after accidental ingestion of tapeworm eggs, produced by adult tapeworms, which reside in the GI tract of definitive hosts. The eggs are shed with the feces of definitive hosts and are thus deposited on vegetation, soil, and/or in water (via runoff from soil), which facilitates entry into the food web. After ingestion, the eggs hatch and release oncospheres (a larval stage of the tapeworm). The larvae exit the intestine by penetrating the wall and enter the bloodstream to reach internal organs (commonly the liver) to which they attach, encyst, and proliferate. Over time, the volume of the cyst increases, while budding and/or metastases lead to the formation of daughter cysts in the same organ or spread to other organs [9]. The disease progresses slowly, eventually leading to organ compression, obstruction of physiological functions, tissue destruction, and impairment or loss of function [7].

Worldwide, nearly all echinococcosis cases are caused by the *E. granulosus s. l.* complex, with 22% estimated to result from *E. canadensis* G6–8,10 and 73% from *E. granulosus sensu stricto* (*s. s.*) G1/3 [10]. Cases of AE are comparatively much more infrequent, due in part to the uneven geographical distribution of *E. multilocularis*, with endemic areas primarily in the northern hemisphere, but also the tapeworm’s patchy local distribution pattern within these areas [9]. Based on a recent systematic review by Casulli et al. in 2023 [7], the mean annual incidence of echinococcosis in the EU was estimated as 0.5 cases per 100,000 people, while the incidence for the entire continent was slightly higher, 0.64 cases per 100,000 (based on cases recorded from 1997–2020). The number of registered cases in Serbia in the last decade has been variable, with most reported in 2017 (>70), and the least in 2020 and 2021 (<10). As the number of cases started rising in 2022, however, and reached 21 in 2023, the drop was likely caused by inefficient reporting during the COVID-19 pandemic [11]. The number of cases may continue to rise, as according to Casulli et al. [7], the Balkan Peninsula could be the current epicenter of CE in Europe. The first confirmed cases of AE in Serbia emerged in the last three years in the northern Vojvodina province [12,13].

In Europe, tapeworms of the *E. granulosus s. l.* complex are primarily thought to cycle domestically, between livestock and dogs [7], while *E. multilocularis* cycles sylvatically between small mammals (rodents) and foxes [8]. Similarly, in Serbia, dogs are considered the primary definitive host of *Echinococcus* spp., which cause human CE [14], while more recently, foxes and especially, golden jackals, have been implicated as important hosts of *E. multilocularis* [15,16,17]. As domestic dogs are a highly versatile species which can be trained to perform specific tasks (hunting, herding) and are able to adapt to different lifestyles, including those with moderate or low dependency on humans for survival (stray dogs), they may contribute to the spillover of *Echinococcus* spp. between domestic and sylvatic environments [2,7].

Despite the importance of echinococcosis to public health, molecular identification of the *Echinococcus* spp. which circulate in dogs in Serbia is currently lacking. The aim of this study was to assess the molecular occurrence of Cyclophillidean tapeworm infections in hunting and stray dogs, and specifically those caused by *E. multilocularis* and *E. granulosus s. l.* complex. This study is part of an ongoing effort to identify definitive hosts that are potential reservoirs of *E. multilocularis* in Serbia.

## 2. Materials and Methods

### 2.1. Fecal Sample Collection

Fecal samples were collected in 2025 from active hunting dogs and strays, recently captured and transported to a veterinary clinic with a temporary shelter. Sampling was based on convenience and contingent upon signed informed consent (for owners and shelter staff) and signed cooperation agreements (for dog shelters). Samples of hunting dogs (n = 29) were retrieved by owners from individual kennels, while the stray dog samples were collected in individual and community kennels as well as a dog-run on the shelter grounds (n = 66). To ensure sample individuality in shared environments at the shelter, samples were collected immediately after defecation. Hunting dogs originated from four locations in eastern Serbia (Šuvajić, Srednjevo, Tribrode, Veliko Gradište) in the Danube valley and one location in central Serbia (Smederevska Palanka). Stray dogs originated from various localities (mostly villages) surrounding the township of Ub, southwest of Belgrade, a farming and agricultural area in which several large abattoirs are located. Based on the information obtained from owners and/or shelter staff at the time of sample collection, none of the dogs were treated with antihelminthics or formulations effective against cestodes prior to sampling. Antihelminthic/anti-cestode drugs were, however, administered to stray dogs after the samples were collected, as per the established checkup and treatment procedure at the veterinary clinic. Owners of hunting dogs were advised to treat their dogs regularly. As per the decision of the institutional ethical review board at the Institute for Medical Research of the University of Belgrade, this study did not require ethical approval, as the samples were collected without intervention. Informed consent was obtained from the owners of the hunting dogs and the management of the shelter for sample collection and publication.

### 2.2. Isolation and Enrichment of Taeniid Eggs from Fecal Samples

To collect taeniid eggs, 5–10 g of fecal material was first mixed and homogenized with 10–15 mL of 3% dPBS-Tween20 solution and strained through nylon mesh strainers with a pore size of 0.5 mm to remove larger particles. The methodology to isolate and enrich for taeniid eggs has been described in Mathis et al., 1996 [18], and Aschenborn et al., 2023 [19]. Briefly, the homogenized feces were centrifuged at 400× *g* for 10 min, the supernatant was discarded, and 15 mL of zinc chloride (ZnCl_2_) solution (1.45 specific gravity) was added to each sample and mixed well. The samples were centrifuged at 1600× *g* for 30 min with the brake off, the supernatant was poured through a mesh filter cloth with a pore size of 50 μm, followed by a mesh filter cloth with a pore size of 22 μm (Franz Eckert GMBH, Waldkirch, Germany). The 22 μm filter was inverted, and taeniid eggs captured in the mesh were washed into a 50 mL centrifuge tube using a squirt bottle filled with type 3 water. The eggs were pelleted by centrifugation and transferred into microcentrifuge tubes. The pellet was finally re-suspended in 100 μL of nuclease-free water.

### 2.3. DNA Extraction and Molecular Detection of Cyclophyllidean, E. granulosus s. l. Complex and E. multilocularis DNA

DNA was extracted from 30 μL of the egg pellet using Trizol reagent according to the manufacturer’s instructions (Invitrogen, Thermo Fisher Scientific, Waltham, MA, USA). Screening for the presence of cyclophyllidean cestode DNA was performed using a nested PCR protocol described by Martini et al., 2022 [20], while a published multiplex real-time qPCR assay was used to detect DNA of the *E. granulosus s. l.* complex [21] and a direct conventional PCR assay for *E. multilocularis* [22]. The detection assays were run for all fecal samples (n = 95). The primer sequences, annealing temperatures, and expected amplicon lengths for the Cyclophyllidea and *E. multilocularis* targets are shown in Table 1. Briefly, each reaction contained 3 μL of the egg pellet DNA as the template, 200 pmol of each primer, and 12.5 μL of 2X Multiplex Hot-Start PCR Master Mix (biotechrabbit, Berlin, Germany) in a total volume of 25 μL. Positive (*E. multilocularis* DNA confirmed by sequencing) and negative (nuclease-free water) controls were included in each run. Assays were run in a Veriti thermocycler (Applied Biosystems, Thermo Fisher Scientific, Waltham, MA, USA). The products were visualized on Agarose gels with EtBr. Each reaction for the detection of DNA of tapeworms of the *E. granulosus s. l.* complex contained 5 μL of the egg DNA as the template, 200 nM of each primer (as a primer mix) and probe, 12.5 μL of 2X Taqman Universal PCR Master Mix (Applied Biosystems, Fisher Scientific, Waltham, MA, USA) in a final volume of 25 μL. The reactions were performed in 96-well plates in a StepOne Plus Real-time PCR cycler (Applied Biosystems, Fisher Scientific, Waltham, MA, USA).

### 2.4. Statistical Analyses

Confidence intervals (95%) for sample proportion/prevalence were calculated using the Epitools Epidemiological Calculators (https://epitools.ausvet.com.au/, accessed on 5 February 2026) [23]. For comparison between groups, chi-square or Fisher’s exact test was performed, as appropriate.

## 3. Results

Cyclophyllidea DNA was detected in 60% (95% CI = 49.9–69.3%) of the fecal samples (n = 95) by PCR screening, while the occurrence of *E. canadensis* G6–8,10 was 4.2% (95% CI = 1.6–10.3%) and of *E. multilocularis* 3.2% (95% CI = 1.1–8.9%). Other species of the *E. granulosus s. l.* complex, as well as co-infections with different *Echinococcus* spp., were not detected. Overall, *Echinococcus* spp. DNA was detected in 7.4% (95% CI = 3.6–14.4%) of the fecal samples (Table 2).

When analyzed per group, Cyclophyllidea DNA was detected in 48.3% (95% CI = 31.4–65.6%) of hunting and 65.1% (95% CI = 53.1–75.5%) of stray dogs. *E. canadensis* G6–8,10 DNA was detected in 6.9% (95% CI = 1.9–22%) of hunting and 3% (95% CI = 0.8–10.4%) of stray dogs. *E. multilocularis* DNA was detected only in stray dogs, 4.6% (95% CI = 1.6–12.5%). A greater overall occurrence of *Echinococcus* spp. DNA, 7.6% (95% CI = 3.3–16.5%), was noted in fecal samples of stray dogs (Table 3).

## 4. Discussion

For this study, 95 fecal samples of dogs (hunting and strays) were examined for the presence of cyclophyllidean DNA and specifically *E. granulosus s. l.* complex and *E. multilocularis* DNA. The applied methodology uses a high-density flotation solution suitable for a variety of parasite eggs/oocysts [24], in combination with mesh filters to allow for efficient isolation and enrichment of taeniid eggs [18,19,25,26]. This protocol is particularly efficient for *Echinococcus* spp. eggs [18,27]. PCR screening was based on primers designed to amplify a fragment of the *COX1* gene of a broad spectrum of cyclophyllidean cestodes [20]. Overall, the results showed that 60% (95% CI = 49.9–69.3%) of the examined fecal samples contained cyclophyllidean DNA (Table 2), detected in 48.3% (95% CI = 31.4–65.6%) of hunting dog and 65.1% (95% CI = 53.1–75.5%) of stray dog samples. The difference was not statistically significant (Table 3). With respect to the protocol used for egg isolation, it stands to reason that the cyclophyllidean DNA detected in the fecal samples is predominantly of Taeniidae origin, while DNA of other cestodes is likely less abundant, but cannot be entirely excluded. *Echinococcus* spp. DNA was detected in 7.4% (95% CI = 3.6–14.4%) of the fecal samples; specifically, *E. canadensis* G6–8,10 DNA was present in both groups of dogs (n = 4 in total), 4.2% (95% CI = 1.6–10.3%), while *E. multilocularis* DNA was present only in stray dogs (n = 3), 4.6%, (95% CI = 1.6–12.5%). To the best of our knowledge, this study is the first to molecularly confirm the presence of these tapeworm species in dogs in Serbia.

### 4.1. Detection of Cyclophyllidean DNA in Fecal Samples

The results of the screening for cyclophyllidean DNA presented here point to a fairly high occurrence of tapeworm infections, which is not entirely consistent with previously published studies from Serbia. A study by Sommer et al. from 2017 determined the presence of taeniid eggs in 1.5% (n = 134) of dogs from private shelters near Belgrade [28], while a recent study from 2021 by Ilić et al. reported taeniid eggs in 1% of dog fecal samples (n = 1267) collected from six public dog shelters in Serbia, with complete absence of taeniid eggs in samples from four shelters [29]. Previously, in 2017, Ilić et al. reported the absence of taeniid eggs in fecal samples (n = 421) of pet dogs from Belgrade [30]. A later study of owned dogs by Jovanović et al., 2024 [31], which also examined hunting dogs, reported taeniid eggs in 1.3% of fecal samples (n = 382). Taeniid eggs were found in 15.6% (n = 282) of dog feces samples collected from public areas in the city of Kruševac [32]. As accidental collection of stray dog feces from public areas cannot be excluded, this may account for the higher occurrence of taeniid eggs reported by Raičević et al. in 2021 [32]. Much earlier studies showed taeniid eggs in the feces of 17.45% owned (n = 95) and of 50% stray dogs (n = 150) from eastern Serbia’s city of Požarevac [33]. A study of the wider area of eastern Serbia by Đurić et al. [34] found taeniid eggs in the feces of 5.26% of shelter dogs (n = 345), while similarly, taeniid eggs were found in 6.6% (n = 151) of dogs from Belgrade [35]. Collectively, these studies indicate that the occurrence of Taeniidae infection in dogs appears to be highly variable. While some variability may be accounted for by inherent differences in origin of the samples and/or specificities associated with the groups of dogs examined, the primary cause for the discrepancy in the findings with respect to this study is likely differences in methodology. The performance (sensitivity) of classical routine diagnostic methods based on conventional flotation (gravitational and/or centrifugal) and visual examination has been reported to be low for taeniid eggs [25,36,37].

### 4.2. Detection of Echinococcus spp. DNA in Fecal Samples

Interestingly, based on a widely adopted practice when performing coprological examinations for veterinary diagnostics, findings of taeniid eggs may be attributed to *Echinococcus* spp. as a safety precaution, without molecular confirmation [31,32]. All of the samples in this study, in which *Echinococcus* spp. DNA was detected, were also found to contain Cyclophillidean DNA, which is not surprising; however, based on the results shown here, the likelihood that a significant proportion of the findings of taeniid eggs on a coprological exam actually represent findings of *Echinococcus* spp. is low. In light of that, the true prevalence of infection of dogs in Serbia with *Echinococcus* spp. may be unknown. The finding of *Echinococcus* spp. DNA suggests that fresh (i.e., not frozen) raw meat was available as a food source, at least for seven dogs (Table 3). The fecal samples of two dogs from each of the examined groups contained *E. canadensis* G6–8,10 DNA, part of the *E. granulosus s. l.* complex. Samples of domestic pigs and wild boar, which contain *E. granulosus s. l.* complex DNA, specifically *E. canadensis* identified as G7 [38] and as G6–8,10 [39], were previously reported in Serbia. *E. canadensis* G6–8,10 DNA was also detected in fecal samples of golden jackals [17]. The combined results suggest that *E. canadensis* G6–8,10 tapeworms circulate domestically and sylvatically in Serbia. Only two human cases of *E. canadensis* G7 infection of Serbian origin, confirmed by molecular methods, were published [40], while another study by Schneider et al. identified nine cases among immigrants from the former Yugoslavia to Austria [41]. On the European level, 88.4% of cases are attributed to *E. granulosus s. s.* G1/3 and 11.1% to *E. canadensis* G6/7 [42]. Despite the lower occurrence in humans, infections by *E. canadensis* genotypes should not be underestimated in the context of public health, especially given that in Serbia, molecular identification of *Echinococcus* spp. from human tissues is not routinely done. Additionally, Serbia is part of a larger geographical hotspot for human CE caused by *E. canadensis* G6/7, which includes several central and eastern European countries [42]. Despite a low occurrence, *E. canadensis* G6–8,10 DNA in the feces of hunting dogs is of particular concern due to the increased probability for contamination of outdoor spaces shared with owners (yards, gardens) with tapeworm eggs, as well as the possibility of direct owner contact with feces containing eggs (cleaning kennels). Raising awareness of owners on the risk of infection and the importance of regular deworming is thus a priority. Of equal importance is continual education of the owners to ensure appropriate disposal and/or destruction of slaughter waste and carcasses, and to prevent deliberate feeding of dogs with fresh, raw meat (offal), as well as addressing predatory and/or independent hunting behavior with appropriate training. A direct and perhaps significant contribution of human activity and/or practice(s) to the spread and transmission of *E. canadensis* G6–8,10 in Serbia cannot be ruled out.

### 4.3. Dogs as Reservoirs of Echinococcus spp. Eggs and Implications for Public Health

The relatively high occurrence of *Echinococcus* spp. DNA in fecal samples of stray dogs, 7.6%, is of concern, especially as DNA of *E. multilocularis* was detected in three dogs (4.6%, 95% CI = 1.6–12.5%) (Table 3). In golden jackals from different parts of Serbia (n = 122), the occurrence of *Echinococcus* spp. DNA was 8.2% (95% CI = 4.2–14.9%), 70% of which (7/10) was *E. multilocularis* [17]. *E. multilocularis* DNA was also detected in the liver of a domestic pig recently [39]. Thus, spillover of *E. multilocularis* into the domestic cycle has occurred, which increases the risk for human infection. These findings are of particular importance to public health, given the severity of AE and its recent emergence in this country. An additional implication of this study is that, in addition to golden jackals, stray dogs also contribute to the maintenance and spread of *E. multilocularis* in Serbia. According to a large national-level study in Poland by Wierzbowska et al., 2016 [43], the incursion of domestic dogs into hunting grounds has a significant ecological impact on wildlife manifested as stock reduction of prey species and competition with other predators. Although similar studies have not been conducted in Serbia, hunting grounds are integrated in the landscape around urban and rural zones, and many are not fenced in, providing access opportunities for free-roaming dogs, both owned and stray. Despite the fact that *E. multilocularis* was not detected in hunting dogs analyzed here, it cannot be excluded that both hunting and stray dogs facilitate the spillover of *E. multilocularis* from hunting grounds. Further studies are needed to assess the frequency of dog incursions into hunting grounds in Serbia. This study examined a relatively small number of samples from a limited geographical area, so the results do not present an accurate determination of the prevalence of *Echinococcus* spp. infection in hunting and stray dogs in Serbia. Nonetheless, the findings suggest that stray dogs are an important reservoir of *E. multilocularis*. Currently, no systematic surveillance and monitoring of definitive hosts for the presence of *E. multilocularis* is in place in Serbia. Based on the evidence from this and other scientific studies, the introduction and implementation of such a program in the future is necessary. Given that stray dogs often roam in and around settlements and urban environments, generally preferring areas in much closer proximity to humans as opposed to golden jackals, the contamination of soil with *E. multilocularis* eggs shed in the feces of stray dogs may thus occur in public parks, playgrounds, gardens, and on agricultural plots. Innovative deworming strategies to specifically target free-roaming stray dogs and to additionally cover hunting grounds may need to be designed for effective *E. multilocularis* transmission control and prevention.

## 5. Conclusions

The findings of this study indicate that dogs participate in the maintenance, spread and possibly transmission of *Echinococcus* spp. tapeworms in Serbia. The detection of *E. multilocularis* DNA in stray dogs is of concern to public health. Given that the Balkans may be the future epicenter of echinococcosis in Europe and that AE is an emerging disease in Serbia, the development of surveillance and monitoring programs for definitive hosts is an urgent matter. Tapeworm species identification by molecular methods needs to be introduced as part of the routine diagnostic workup for reported human and animal cases. Further studies to gain a better understanding of the current epidemiological situation of *E. multilocularis* are necessary, while the development of innovative transmission control and prevention strategies should be considered. Specific educational campaigns should be implemented immediately to raise awareness of echinococcosis, transmission routes and major sources of infection for definitive hosts, as well as the importance of regular deworming of dogs. The involvement of veterinarians and organizations aimed at improving animal health, as well as protection, welfare and adoption of stray dogs is crucial in this effort.

## Figures and Tables

**Table 1:** Primer sequences, annealing temperatures, and expected amplicon lengths for conventional PCR.

Primer (5′ → 3′)	Annealing Temperature	Expected Amplicon
Cyclo forward: TTTGATCGTAAATTTAGTTCTGC Cyclo reverse: GCAACAACAAATCAAGTATCATG Cyclo nested forward: GTTCTGCTTTTTTTGATCC Cyclo nested reverse: GTATCATGTAGAACTTTATC [20]	50 °C	466 bp
EM-H15: CCATATTACAACAATATTCCTATC EM-H17: GTGAGTGATTCTTGTTAGGGGAAG [22]	53 °C	200 bp

**Table 2:** Proportion of Cyclophyllidea, *E. canadensis* G6–8,10 and *E. multilocularis* DNA of the total number of fecal samples (n = 95).

PCR Screening Target	Proportion (95% CI)
Cyclophyllidea	60% (49.9–69.3%)
*E. canadensis* G6–8,10	4.2% (1.6–10.3%)
*E. multilocularis*	3.2% (1.1–8.9%)
**Total** *Echinococcus* spp.	7.4% (3.6–14.4%)

**Table 3:** Number, proportion, and 95% CI of hunting and stray dog fecal samples in which Cyclophyllidea, *E. granulosus s. l.* and *E. multilocularis* DNA were detected, and comparison between groups.

Dogs	Cyclophyllidea	*E. canadensis* G6–8,10	*E. multilocularis*	*Echinococcus* spp. Total
Hunting	14 (48.3%, 95% CI = 31.4–65.6%)	2 (6.9%, 95% CI = 1.9–22%)	0 (0%, 95% CI = 0–11.7%)	2 (6.9%, 95% CI = 1.9–22%)
Stray	43 (65.1%, 95% CI = 53.1–75.5%)	2 (3%, 95% CI = 0.8–10.4%)	3 (4.6%, 95% CI = 1.6–12.5%)	5 (7.6%, 95% CI = 3.3–16.5%)
***p*-value**	0.122 ^1^	0.583 ^2^	0.551 ^2^	0.907 ^2^

^1^ Chi-square test. ^2^ Fisher’s exact test.

## Data Availability

All the original data is available from the corresponding author.
